# An Ultrasensitive Molecularly Imprinted Point‐Of‐Care Electrochemical Sensor for Detection of Glial Fibrillary Acidic Protein

**DOI:** 10.1002/adhm.202401966

**Published:** 2024-09-02

**Authors:** Yixuan Li, Liuxiong Luo, Lenart Senicar, Rica Asrosa, Burcu Kizilates, Kaizhong Xing, Elias Torres, Lizhou Xu, Danyang Li, Neil Graham, Amanda Heslegrave, Henrik Zetterberg, David J. Sharp, Bing Li

**Affiliations:** ^1^ Institute for Materials Discovery Department of Chemistry University College London London WC1E 7JE UK; ^2^ School of Materials Science and Engineering Central South University Changsha 410083 P. R. China; ^3^ Yusuf Hamied Department of Chemistry University of Cambridge Cambridge CB2 1EW UK; ^4^ Graphenea Semiconductor Paseo Mikeletegi 83 San Sebastián 20009 Spain; ^5^ College of Biosystems Engineering and Food Science Zhejiang University Hangzhou 310058 China; ^6^ ZJU‐Hangzhou Global Scientific and Technological Innovation Center Zhejiang University Hangzhou 311215 P. R. China; ^7^ Research Center The Seventh Affiliated Hospital Sun Yat‐sen University Shenzhen 518107 P. R. China; ^8^ Department of Brain Sciences Imperial College London London W12 0BZ UK; ^9^ UK Dementia Research Institute at UCL University College London London WC1E 6BT UK; ^10^ Department of Neurodegenerative Disease UCL Institute of Neurology London WC1E 6BT UK; ^11^ Department of Psychiatry and Neurochemistry Institute of Neuroscience and Physiology The Sahlgrenska Academy at the University of Gothenburg Mölndal S‐431 80 Sweden; ^12^ Clinical Neurochemistry Laboratory Sahlgrenska University Hospital Mölndal S‐431 80 Sweden; ^13^ Hong Kong Centre for Neurodegenerative Diseases Hong Kong 999077 P. R. China; ^14^ Wisconsin Alzheimer's Disease Research Center University of Wisconsin School of Medicine and Public Health University of Wisconsin‐Madison Madison WI 53792 USA; ^15^ Care Research & Technology Centre UK Dementia Research Institute London W12 0BZ UK

**Keywords:** glial fibrillary acidic protein, molecularly imprinted polymer, neurological disease monitoring, point‐of‐care electrochemical sensor, reduced graphene oxide

## Abstract

Accurate assessment of neurological disease through monitoring of biomarkers has been made possible using the antibody‐based assays. But these assays suffer from expensive development of antibody probes, reliance on complicated equipments, and high maintenance costs. Here, using the novel reduced graphene oxide/polydopamine‐molecularly imprinted polymer (rGO/PDA‐MIP) as the probe layer, a robust electrochemical sensing platform is demonstrated for the ultrasensitive detection of glial fibrillary acidic protein (GFAP), a biomarker for a range of neurological diseases. A miniaturized integrated circuit readout system is developed to interface with the electrochemical sensor, which empowers it with the potential to be used as a point‐of‐care (POC) diagnostic tool in primary clinical settings. This innovative platform demonstrated good sensitivity, selectivity, and stability, with imprinting factor evaluated as 2.8. A record low limit‐of‐detection (LoD) is down to 754.5 ag mL^−1^, with a wide dynamic range from 1 to 10^6^ fg mL^−1^. The sensing platform is validated through the analysis of GFAP in clinical plasma samples, yielding a recovery rate range of 81.6–108.8% compared to Single Molecule Array (Simoa). This cost‐effective and user‐friendly sensing platform holds the potential to be deployed in primary and resource‐limited clinical settings for the assessment of neurological diseases.

## Graphical abstract

A novel reduced graphene oxide/polydopamine‐molecularly imprinted polymer (rGO/PDA‐MIP) point‐pf‐care (POC) sensing platform for the detection of glial fibrillary acidic protein (GFAP) is developed, which shows remarkably high sensitivity, selectivity, robustness, and record limit‐of‐detection (LoD) at attomolar level with expansive detection range, making it a promising approach for screening for neurological diseases and monitoring progression.

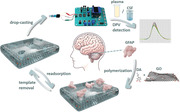

## 1 Introduction

Assessment of neurological diseases, such as dementia, is complicated by several factors.^[^ ^1^ ^]^ These include: the mild or no symptoms at the early stage, which makes the conventional symptom‐based examination challenging; the limited access to current gold standard tests, such as positron emission tomography or cerebrospinal fluid (CSF) analysis through spinal lumbar puncture, which are expensive and requiring highly skilled clinical staff, and variable in practitioner experience. These factors produce long waiting time, delayed diagnosis and intervention. An estimated 30% of people with dementia never receive a formal diagnosis.^[^ ^2^ ^]^ Recently, great progress has been made in the assessment of neurological diseases through the monitoring of body fluid biomarkers^[^ ^3^, ^4^ ^]^ For example, phosphorylated tau 217 in blood has been found as one of the most promising biomarkers of the emerging Alzheimer's disease.^[^ ^5^ ^]^ This is of great importance as it would allow more accessible and equitable life‐changing interventions in time, thereby substantially enhancing the life quality for affected individuals.^[^ ^6^ ^]^ However, the current commercial technologies for precise determination of these biomarkers largely rely on the advanced antibody‐based assays, for example, Single Molecule Array (Simoa), enzyme‐linked immunosorbent assays (ELISA),^[^ ^7^ ^]^ and electrochemiluminescence,^[^ ^8^ ^]^ which require development of expensive new antibodies as assay probes, complicated fluorescent labeling, demanding laser excitation and signal capture systems, as well as highly skilled operational personnel and high maintenance cost.^[^ ^5^, ^9^ ^]^ Emerging technologies have been developed in well‐equipped laboratories for biomarker analysis. For example, an impedimetric immunosensor has been developed for ultrasensitive detection of glial fibrillary acidic protein (GFAP) in serum with the LoD of 51.0 fg mL^−1^;^[^ ^10^ ^]^ an electrochemical skin sensor for simultaneous measurement of GFAP and Interleukin‐6;^[^ ^11^ ^]^ a graphene‐based solution‐gated field‐effect transistor sensor for label‐free test of phosphorylated tau 217;^[^ ^12^ ^]^ ratiometric fluorescence sensing technique for amyloid β peptide measurement.^[^ ^13^ ^]^ However, these technologies still suffer from the disadvantages of protein‐based probes (high development cost, high batch‐to‐batch difference, strict requirement to analytical environment), inadequate limit‐of‐detection (LoD) for detecting ultratrace analytes, and lack of validation in patient samples (with naturally produced target biomarkers), which significantly restrict the point‐of‐care (POC) application of these methods in primary healthcare environments and health screening initiatives.

Molecularly imprinted polymer (MIP) is a polymeric matrix that features cavities mimicking antibody‐antigen interactions for specific template binding.^[^ ^14^ ^]^ Recent advancements have shown the development of MIP‐based sensors for the ultrasensitive detection of a variety of proteins, demonstrating exceptional sensitivity and selectivity in clinical sample analysis.^[^ ^15^ ^]^ Compared with biological probes‐based assays for proteins detection, MIP shows the advantages of low cost, ease of utilization, long‐term stability, high reproducibility, and reusability, also possessing the potential to be integrated with micro sensing platforms, for example, electrochemical sensors, field‐effect transistor sensors, etc, to constitute POC devices. Nonetheless, traditional MIP‐based sensors exhibit inherent drawbacks for proteins detection. For instance, the MIP monomers tend to be dense, making it difficult for macromolecular templates (proteins) to reach (or leave) established cavities, which would lead to poor molecular transmission and permanent entrapment.^[^ ^16^ ^]^ The traditional non‐physiological fabrication condition can also be problematic, since it often denature or aggregate the proteins or change their conformation, leading to the deformed imprinting cavities, which would degrade the specificity of detection.^[^ ^17^ ^]^ In addition, the cavities heterogeneity of MIP shows great impact on the detection of proteins, as it might cause the non‐specific combination of recognition cavities with interfering proteins that have similar shapes to the target proteins or flexibility that can partially fit in the imprinting sites.^[^ ^18^ ^]^ These issues would potentially lead to the diminished performance of MIP‐based sensors for the recognition of proteins, which might explain the scarce reports on MIP sensor applications for protein detection with superior performance.^[^ ^19^ ^]^

Here we report a novel POC sensing platform using reduced graphene oxide/polydopamine (rGO/PDA)‐MIP matrix for the detection of GFAP, which is an astrocytic cytoskeletal protein and a discriminative biomarker for a range of neurological diseases,^[^ ^20^, ^21^, ^22^, ^23^, ^24^ ^]^ including traumatic brain injury, glioblastoma multiforme, multiple sclerosis, stroke, and Alzheimer's disease.^[^ ^25^ ^]^ For healthy humans, GFAP at low concentration (30‐70 pg/mL) could be detected in CSF and plasma. However, for the patients with neurological diseases, this concentration would increase to a much higher range.^[^ ^26^ ^]^ Therefore, the development of a sensing technique with an ultralow LoD and wide dynamic range becomes crucial. The PDA‐MIP layer is synthesized onto graphene oxide (GO) through a novel molecular imprinting technology, effectively increasing the number of accessible imprinting cavities. We have coupled this electrochemical sensor with a home‐designed miniaturized readout system with function of differential pulse voltammetry (DPV), making it possible to be used by the primary practitioners and patients as a POC platform. This sensing platform has been validated in human plasma and CSF, demonstrating the superior performance over conventional ELISA with LoD down to attomolar level, which makes it a promising tool for neurological diseases assessment.

## 2 Results and Discussion

### 2.1 Overview of rGO/PDA‐MIP POC Sensing Platform

**Figure 1:**
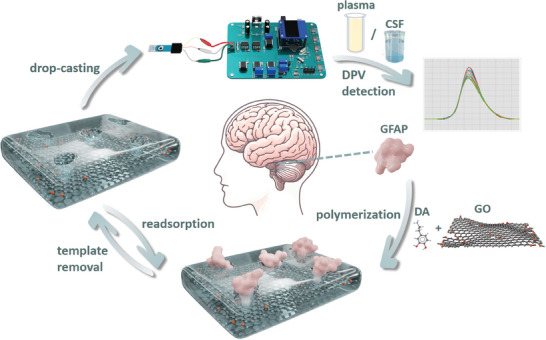
Illustration of the synthesis of surface‐MIP composite and its application in the detection of GFAP using the home‐designed POC readout platform.

In this project, based on the imprinting technology, we developed a rGO/PDA‐MIP‐modified electrochemical sensor interfaced with a miniaturized circuit readout system (Figure S1, Supporting Information) for the POC detection of GFAP, as shown in **Figure** **1**. Dopamine hydrochloride (DA), which serves as the functional monomer, forms hydrogen bonds with GFAP templates to create monomer‐template complex.^[^ ^27^ ^]^ The addition of GO dispersion and oxidant ammonium persulfate (AP) initiates the physical adhesion of this complex onto GO surface^[^ ^28^ ^]^ and its polymerization to form a surface‐PDA‐MIP layer, encapsulating GFAP templates within the polymer matrix with basic overall structure remained.^[^ ^29^, ^30^, ^31^ ^]^ During polymerization, GO would be partially reduced to rGO,^[^ ^32^ ^]^ while DA undergoes oxidation to quinone, followed by the formation of leucodopamine‐chrome through intramolecular cyclization.^[^ ^33^ ^]^ The template molecules are then eluted, leaving behind cavities with size, shape, surface charge distribution, etc, complementary to the target molecules, thus endowing the rGO/PDA‐MIP composite with capability for highly specific GFAP recognition. This strategy would mitigate the cavities heterogeneity to a certain extent, so resulting in higher selectivity. Also, within a certain range, the thinner imprinting layer could reduce the permanent entrapment of protein templates and improve the mass transfer efficiency of template/target molecules,^[^ ^34^ ^]^ leading to a wider detection range. Besides, the mild synthetic environment could effectively avoid the denaturation of protein templates to ensure the highly specific recognition of targets.^[^ ^35^ ^]^ In addition, the DA polymerization process induces a transition of GO into rGO,^[^ ^33^ ^]^ enhancing the electrochemical activity of this PDA‐MIP matrix, which synergizes with the increased surface area to facilitate the rapid electron transfer, thereby leading to the record low LoD for GFAP detection.

### 2.2 Optimization of rGO/PDA‐MIP Composite

A systematic optimization has been carried out to identify the key factors that can affect the performance of rGO/PDA‐MIP sensor, for example, the mass ratio of templates to monomers and the incubation time of the modified electrodes in analyte solution. We have found that the mass ratio of templates to monomers plays a critical role in determining the number of imprinted cavities within the polymer matrix, which can significantly influence the current change (Δ*I*) (peak current difference in the absence and presence of the analyte) during detection, and thereby affecting the sensitivity and the detection range. As illustrated in Figure S2A (Supporting Information), with the fixed amount of DA monomer at 2 mg, variations in GFAP template amount result in the notable changes in Δ*I*, with an optimal peak observed at 1.5 µg of template, indicating maximal recognition capability of the rGO/PDA‐MIP composite toward the target molecules. This is attributed to that the deficient template leads to a reduced number of imprinting cavities, whilst the excessive template hampers monomer polymerization, in turn leading to the decreased number of binding cavities and even ineffective imprinting layer.^[^ ^36^ ^]^

The impact of incubation time on the response of MIP sensor was also explored. To avoid the cross‐influence, the effect of different incubation time on MIPs which fabricated with different amount of templates was also explored. Figure S2B (Supporting Information) investigated the change of Δ*I* versus the change of incubation period of MIPs modified electrode in 1 pg mL^−1^ GFAP solution. When the coated MIP was fabricated with addition of templates at 1.5 µg, the maximum increase in Δ*I* caused by target recognition has been observed, which saturated after the 10 min mark. This indicated that GFAP targets initially bind rapidly with the imprinting cavities, eventually at ≈10 min reaching a dynamic equilibrium between adsorption and desorption processes. When coated MIPs were fabricated with templates at 1.0 or 2.0 µg, Δ*I*s changed less with optimal incubation time slightly altered, but still showed similar increasing trend with increasing incubation time. Differently, as for the groups with MIPs prepared with templates at 2.5 and 3.0 µg, Δ*I*s kept low even if the incubation time increased, which demonstrated that MIPs fabricated under these conditions possessed no target recognition capability. This could be due to the excessive templates leading to the failure of monomers polymerization, which further led to the ineffective imprinting layer.^[^ ^37^ ^]^

### 2.3 Characterization of rGO/PDA‐MIP Composite

**Figure 2:**
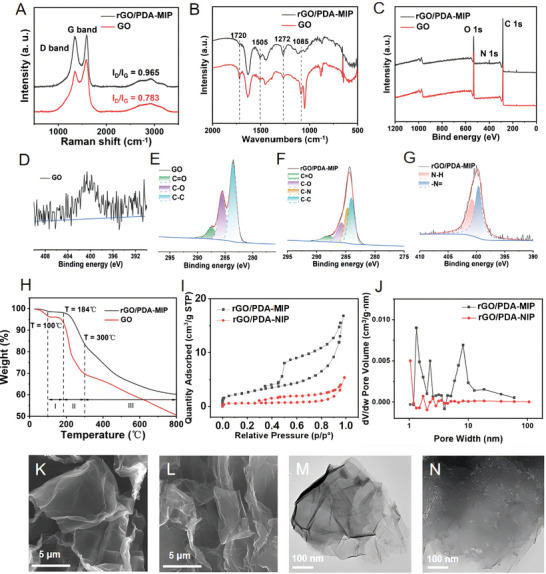
Characterization of GO, rGO/PDA‐MIP, and rGO/PDA‐NIP composites. A) Raman spectra, B) FTIR spectrum, C) XPS spectra of GO and rGO/PDA‐MIP composite, with XPS N1s spectra of D) GO and G) rGO/PDA‐MIP composite, and XPS C1s spectra of E) GO and F) rGO/PDA‐MIP composite; H) TGA plot of GO and rGO/PDA‐MIP composite; I) Nitrogen adsorption‐desorption isotherm of of rGO/PDA‐MIP and NIP composites with J) corresponding pore size distribution; SEM images of K) GO, L) rGO/PDA‐MIP composite; TEM images of M) GO, N) rGO/PDA‐MIP composite.

To verify the successful encapsulation of PDA on GO, a series of characterizations had been conducted on the composite, including Raman Spectroscopy, Fourier Transform Infrared Spectroscopy (FTIR), and X‐ray Photoelectron Spectroscopy (XPS). Raman analysis, as shown in **Figure** **2**A, displayed two distinct bands for GO: the D band at 1344 cm^−1^, associated with *sp3*‐hybridized carbon atoms; and the G band at 1570 cm^−1^, related to the E_2g_ vibration of *sp2*‐hybridized graphitized carbon.^[^ ^38^ ^]^ The ratio of D peak to G peak (I_D_/I_G_) increased from 0.783 to 0.965 following PDA encapsulation, indicating the cleavage of oxygen‐containing groups on GO during polymerization.^[^ ^39^, ^40^ ^]^ Additionally, FTIR spectra, as shown in Figure 2B, revealed a reduction of oxygenated peaks after PDA coating, notably the C─O peak at 1085 cm^−1^ and the C═O peak at 1720 cm^−1^. The peaks at 1272 and 1505 cm^−1^, corresponding to the C─N and N─H bonds, were indicative of the amide group in PDA, further confirming successful encapsulation of PDA.^[^ ^41^ ^]^

XPS was utilized to prove the findings above. Figure 2C compares the C1s, N1s, and O1s spectra of GO and the rGO/PDA‐MIP composite. Typically, charging correction is performed by selecting one peak with a known binding energy. However, when multiple elements are involved, multiple reference values are needed to ensure the accuracy of the correction. Therefore, multiple reference peaks (C1s and N1s) were used here for charging correction in XPS data, aimed to precisely correct the overall charging effect of the samples. C1s line is observed at 284.2 eV–the standard reference value for the carbon C1s peak, which is usually derived from graphitized carbon. N1s line is observed at 399.7 eV, which is generally considered to be the standard reference value for the nitrogen N1s peak. This value could derive from nitrogen in polymers and organic compounds which contain amine and/or imine group.^[^ ^42^ ^]^ Briefly, GO lacked N element, as evidenced in the XPS N1s spectrum (Figure 2D). The C1s spectrum of GO (Figure 2E) displayed peaks at 283.5, 285.6, and 287.6 eV, corresponding to C─C, C─O, and C═O bonds, respectively.^[^ ^43^ ^]^ In contrast, the C1s spectra of rGO/PDA‐MIP composite (Figure 2F) showed decreases in C‐O and C = O peaks intensity and the emergence of C─N peak at 284.6 eV, which indicated the reduction of GO during PDA polymerization and the formation of the polymer layer.^[^ ^44^ ^]^ The N1s spectra of the rGO/PDA‐MIP composite (Figure 2G) revealed a prominent peak ≈399.7 eV, decomposable into two Gaussian‐fitted peaks: one at 400.8 eV corresponding to the N─H group from PDA's amine group, and another at 399.7 eV indicative of ─N═ species from the pyridine‐like structure of PDA.^[^ ^45^ ^]^ These characterizations collectively confirmed the successful coating of the PDA layer on GO and the partial reduction of GO to rGO.

Thermogravimetric analysis (TGA) of both GO and the rGO/PDA‐MIP composite was depicted in Figure 2H, covering a temperature range from 20 to 800 °C. GO exhibited a final weight loss of ≈50%, primarily due to the decomposition of volatile oxygen and the pyrolysis of carbocyclic rings.^[^ ^46^ ^]^ In contrast, the pyrolysis of the rGO/PDA‐MIP composite occurred in three stages: initial water vapor loss (stage I), followed by degradation of oxygen‐containing functional groups (e.g., ─COOH, ─COH, and ─C═O) starting at ≈184 °C (stage II), and final stage from 300 to 800 °C (stage III), predominated by the weight loss from both PDA pyrolysis and further decomposition of carbocyclic rings. The final weight loss for the rGO/PDA‐MIP composite was ≈38% which is less than that of GO, indicating enhanced thermal stability compared to GO.^[^ ^47^ ^]^

To validate the successful imprinting of templates, the pore size distribution of rGO/PDA‐MIP and NIP composites were analyzed using the Brunauer‐Emmett‐Teller (BET) method based on nitrogen adsorption. (NIP referred to the composite synthesized under similar conditions as MIP, but in the absence of template molecules addition.) As shown in Figure 2I, the obtained adsorption‐desorption curves exhibited hysteresis loops within the relative pressure (P/P_0_) range of 0.4–0.9, indicating the mesoporous structure of the materials.^[^ ^48^ ^]^ The curve shape, which is consistent with the H4‐type loop, suggested the presence of narrow slit‐like pores, which is the characteristic of the lamellar structure of rGO/PDA‐MIP composite. In contrast, the smaller hysteresis loop of rGO/PDA‐NIP composite indicated fewer cavities.^[^ ^49^ ^]^ Figure 2J presented the distribution of pore size using the Barrett‐Joyner‐Halenda (BJH) method, which further confirmed the porous nature of the MIP. A notable peak at ≈9.19 nm, which is corresponding to the average distance between cavity walls and indicative of the size of imprinted cavities, affirmed the successful GFAP imprinting. Peaks ≈2.10 and 4.18 nm in both MIP and NIP composites were deemed as the false peaks, which are attributed to factors such as internal tension strength, pore connectivity, diversity in pore types, and pore size dispersion.^[^ ^50^ ^]^

The morphological characteristics, as observed through Scanning Electron Microscopy (SEM) and Transmission Electron Microscopy (TEM), provided more direct evidences supporting the inferences from above characterizations. Figure 2K displayed GO with a typical curved layered structure and a relatively smooth surface. After the PDA encapsulation, as shown in Figure 2L, the surface of MIP composite appeared rougher and more blurred. Furthermore, the TEM image of GO (Figure 2M) contrasted markedly with that of rGO/PDA‐MIP composite (Figure 2N), where the latter showed a considerable number of mesopores on the surface, confirming the successful imprinting process.^[^ ^51^, ^52^ ^]^

### 2.4 Electrochemical Behavior of rGO/PDA‐MIP‐Modified Electrode

**Figure 3:**
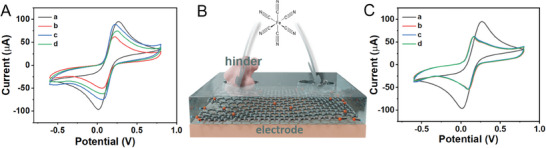
Electrochemical behavior of the rGO/PDA‐MIP and rGO/PDA‐NIP‐modified electrodes. A) CV curves of rGO/PDA‐MIP‐modified electrode, where curve a: bare electrode, curve b: composite‐modified electrode before and curve c: after template elution, curve d: incubation with 1 pg mL^−1^ GFAP; B) Schematic illustration of the mechanism of the electrochemical behavior of rGO/PDA‐MIP‐modified electrode; C) CV curves of rGO/PDA‐NIP‐modified electrode, where curve a: bare electrode, curve b: composite‐modified electrode before and curve c: after template elution, curve d: incubation with 1 pg mL^−1^ GFAP.

Cyclic Voltammetry (CV) had been used to investigate the electrochemical behavior of the MIP‐modified electrode. **Figure** **3**A presented the CV characteristic of the rGO/PDA‐MIP‐modified electrode in presence of [Fe(CN)_6_]^3−/4−^ redox probes. A noticeable reduction in peak current was observed following the encapsulation of the electrode surface with the rGO/PDA‐MIP layer. This decrease was attributed to the layer impeding the access of [Fe(CN)_6_]^3−/4−^ ions to the electrode surface, thereby hindering redox currents. The subsequent removal of GFAP templates had led to an increase in the redox current, indicating the decrease in the resistance of rGO/PDA‐MIP composite. The introduction of the GFAP target at a concentration of 1 pg mL^−1^ again resulted in a decrease in redox current, due to the reoccupation of imprinted cavities within the MIP layer, which in turn reducing the electron transfer between the redox probes and the electrode surface^[^ ^53^ ^]^, as illustrated in Figure 3B.

As a comparative analysis, the electrochemical behavior of the rGO/PDA‐NIP‐modified electrode had also been examined, as depicted in Figure 3C. Similar to the rGO/PDA‐MIP‐modified electrode, encapsulation of the electrode surface with the rGO/PDA‐NIP composite had led to a notable reduction in the redox current. However, unlike the MIP‐modified electrode, neither the elution process nor the incubation with the GFAP target could induce significant changes in the redox current from the NIP‐modified electrode. The lack of responses could be attributed to the absence of imprinted cavities in the rGO/PDA‐NIP layer, suggesting its inability to specifically recognize GFAP targets.^[^ ^36^ ^]^ These observations reinforced the conclusion that the rGO/PDA‐MIP‐modified electrode possesses distinct capability for the detection of GFAP target molecules.

### 2.5 Determination of Sensing Performance with POC Readout System

**Figure 4:**
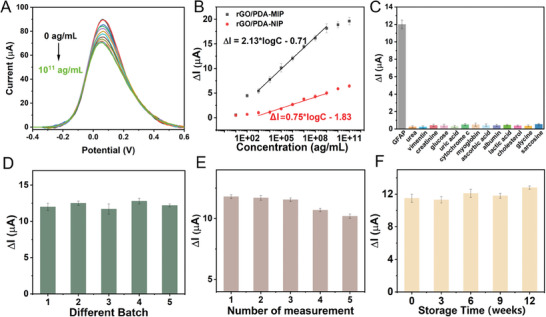
Electrochemical performance of rGO/PDA‐MIP‐modified electrode for the detection of GFAP using the POC readout system. A) The DPV responses of rGO/PDA‐MIP‐modified electrode to different concentrations of GFAP (0, 10^1^, 10^2^, 10^3^, 10^4^, 10^5^, 10^6^, 10^7^, 10^8^, 10^9^, 10^10^, and 10^11^ ag mL^−1^) in the electrolyte; B) Calibration plot of the ∆*I* values versus the concentration of GFAP in the electrolyte tested with rGO/PDA‐MIP and rGO/PDA‐NIP‐modified electrodes respectively; C) The values of ∆*I* to the presence of GFAP and other interferents in equivalent concentration; D) The values of ∆*I* recorded with five batches of rGO/PDA‐MIP‐modified electrodes under same fabrication procedure; E) The values of ∆*I* detected in five repeated measurements with rGO/PDA‐MIP‐modified electrode; F) The values of ∆*I* tested in three months with rGO/PDA‐MIP‐modified electrode. Data are shown as the mean ± SD (*n* = 3).

The rGO/PDA‐MIP‐modified electrode had been integrated with a home‐designed POC readout system for the detection of GFAP with varying concentrations. As illustrated in **Figure** **4**A, the DPV peak currents decreased with the increase of GFAP concentrations. The calibration plot was shown in Figure 4B, which indicated that ∆*I* correlates linearly with the logarithmic GFAP concentration across the range of 10^3^–10^9^ ag mL^−1^ (1–10^6^ fg mL^−1^) with a correlation coefficient of 0.998. The accuracy of this calibration had been verified through triplicate measurements, resulting in relative standard deviation (RSD) <9.0% for each concentration level. The sensitivity of the sensor (slope of calibration line) was determined to be 2.13 µA/log(ag mL^−1^), with LoD determined to be 754.5 ag mL^−1^ (S/N = 3).

Compared to other GFAP electrochemical sensors (referenced in Table S1, Supporting Information), our sensor demonstrated a notably low LoD and a broad detection range. In addition, as for comparison, the calibration plot of rGO/PDA‐NIP‐modified electrode was also presented. It could be seen that the interaction between GFAP and NIP is observable but slight, which could be contributed to a certain degree of non‐specific adsorption of analyte to polymer surface.^[^ ^54^ ^]^ This is mainly caused by the affinity of protein to solid surface except for imprinting sites, depending on electrostatic interaction, hydrophobic interaction, Van der Waals Forces, etc.^[^ ^55^ ^]^ Imprinting factor, the main parameter to evaluate the success of imprinting process, is determined by comparing the amount of target analyte bound by MIP and NIP. It could be calculated based on the below equation^[^ ^56^ ^]^:

(1) $$\mathrm{Imprinting}\;\mathrm{factor}=\frac{Sensitivity_{MIP}}{Sensitivity_{NIP}}$$

The value was therefore assessed to be 2.8, reflecting its good affinity for the target molecule.

The selectivity of the rGO/PDA‐MIP composite‐modified electrode was also evaluated. Cytochrome c (12 kDa), myoglobin (16.7 kDa), albumin (66.5 kDa), and vimentin (54–57 kDa) were selected to evaluate the influence on sensor response from interfering proteins with different sizes. The former two were mainly considered because of their electroactive property, which might cause the current change to some extent. As for albumin, since it serves as the most abundant protein in both plasma and CSF, it is necessary to explore whether it would affect the sensor response before practical application. Worthnotingly, vimentin shares both similar size and typical structural organization of intermediate filaments with GFAP (50 kDa), which makes it most suitable to be applied for the evaluation of MIP's specific target recognition capability. To rigorously assess the selectivity of rGO/PDA‐MIP sensor, except for interfering proteins, a number of common small molecule interferents in human body fluids had also been introduced into the electrolyte. Glycine and sarcosine, as two common amino acids, were first selected. Creatinine, lactic acid, uric acid, and ascorbic acid were introduced considering their electroactivity. Besides, urea, glucose, and cholesterol were also tested due to their abundance in biological samples. To ensure a fair comparison of the sensor response to an equal number of molecules, all interferents including both small molecules and macromolecules were at same concentration with target analyte (0.02 pM). As shown in Figure 4C, the ∆*I* measured by rGO/PDA‐MIP sensor for GFAP detection was significantly higher than those for other interferents. The highest ∆*I* recorded from interferents, which was measured from sarcosine, had been found only 4.5% of the signal for GFAP. The interference raised from substances either with potential electroactivity or with abundance in real body fluids showed with signal change <4.2%. As for vimentin, the interferent requiring the most attention, caused only 2.0% of sensor response change. (The detailed responses from interferents are shown in Table S2, Supporting Information) These findings demonstrated that our rGO/PDA‐MIP sensor has outstanding selectivity for the recognition of GFAP.

Reproducibility, indicated by batch‐to‐batch consistency, was assessed across five batches of the rGO/PDA‐MIP composite‐modified electrodes. The RSD of *I_0_* (peak current in absence of analyte) tested with five batches of MIP modified electrodes is <5.4%, demonstrating the remarkable reproducibility of the electrode modification procedure. What is more, as shown in Figure 4D, the evaluation of ∆*I* further revealed notable reproducibility of the fabricated MIP, with RSD <7.2%. Additionally, the reuse potential of the rGO/PDA‐MIP composite‐modified electrode was tested, as depicted in Figure 4E. A continuous decrease in ∆*I* was observed, likely due to the collapse of few recognition cavities after repeatedly desorbed (polymer change), or the incomplete removal of GFAP molecules after each elution.^[^ ^57^ ^]^ Furthermore, after three months of storage at room temperature, the initial response of the electrode coated with rGO/PDA‐MIP still retained 105.2% of its initial efficacy, as reported in Figure 4F, indicating exceptional long‐term stability. To evaluate the precision of the POC readout system, its performance was compared against a commercial potentiostat (Autolab), as outlined in Figure S3 (Supporting Information). The comparison, illustrated in Figure S4 (Supporting Information), showed an obvious consistency between the peak currents measured by the POC readout system and the Autolab, with a correlation coefficient of 0.982. These detection results demonstrated that the POC readout system possesses reliability comparable to larger, more cumbersome commercial electrochemical workstation.

### 2.6 Detection in Human Body Fluids Samples with POC Readout System

**Figure 5:**
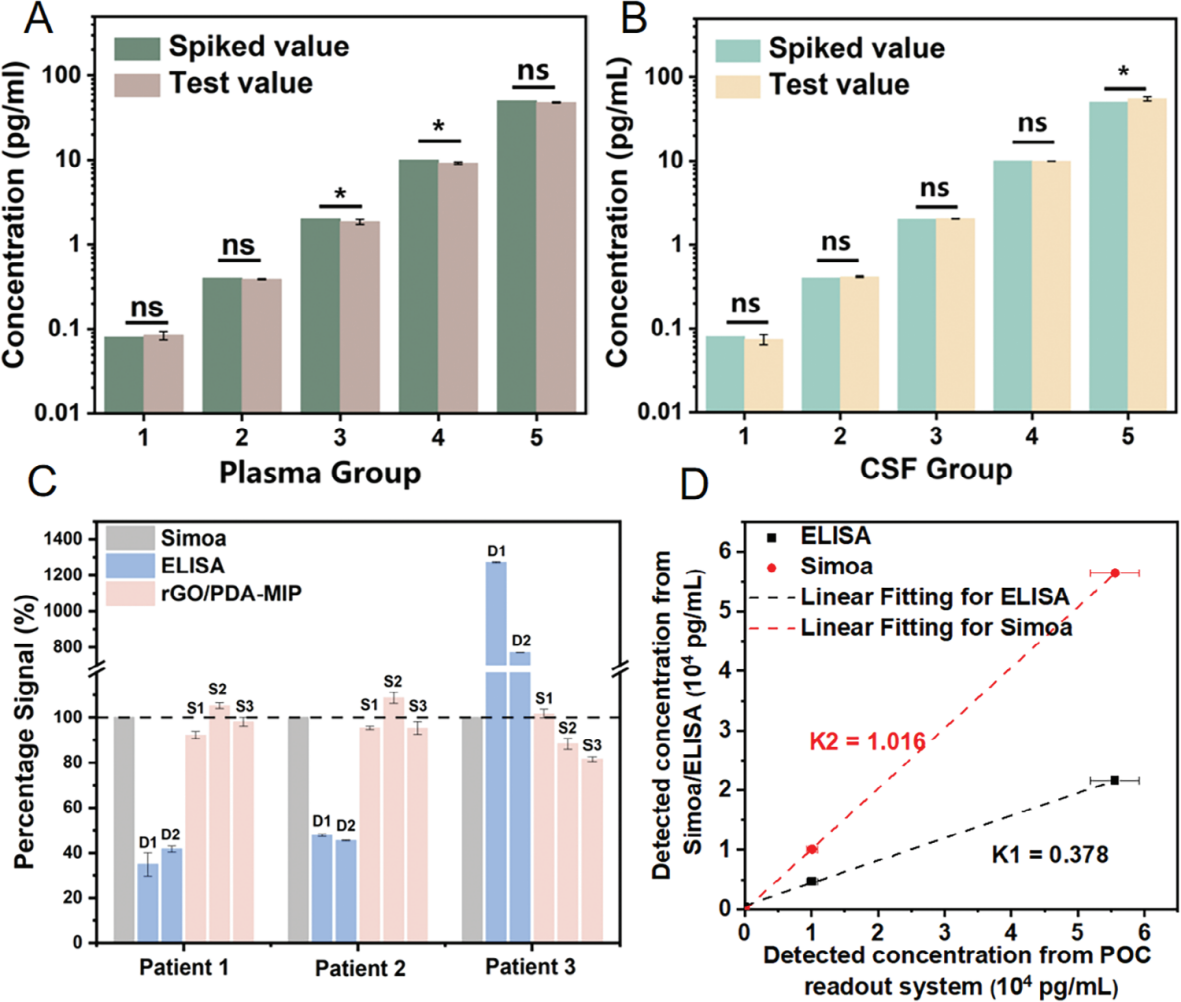
Validation of GFAP detection in human body fluids samples with POC readout system. Comparison between GFAP spiked values and the values tested by this rGO/PDA‐MIP sensor in A) plasma and B) CSF in five sets. C) Signal percentage of GFAP concentration in clinical plasma samples measured by rGO/PDA‐MIP sensor and ELISA in comparison to Simoa as a reference. D) The linear correlation of GFAP concentration in clinical plasma samples measured by the POC readout system versus Simoa and ELISA, respectively. Data are shown as the mean ± SD. *p < 0.05; ns, not significant (*n* = 3).

The importance of closely monitoring GFAP levels in human body fluids lies in its utility as a biomarker for charting the progression of neurological disease. Consistent, accurate monitoring can provide invaluable insights into disease dynamics and response to treatment. Thus, to assess the accuracy and reliability of our sensing method, a series of experiments were first performed to measure GFAP concentrations in spiked human plasma and CSF samples, all conducted in triplicate. As shown in **Figure** **5**A,B, a total of five distinct groups of healthy control plasma and CSF samples were prepared. The spiked concentration difference of GFAP between each group is five times, ranging from a minimal 0.08 pg mL^−1^ to a maximum of 50 pg mL^−1^. Compared with the spiked GFAP value in bodily fluids, no significant difference in test values could be observed for the GFAP detection in plasma at concentrations of 0.08, 0.4, and 50 pg mL^−1^, and in CSF at concentrations of 0.08, 0.4, 2, and 10 pg mL^−1^, respectively; while the RSD of detections showed differences up to 9.6%. The tested recovery rate ranges from 92.3% to 109.6% (Table S4, Supporting Information), which indicated the good affinity and selectivity of the fabricated rGO/PDA‐MIP composite.

To evaluate the clinical applicability of our approach, the developed rGO/PDA‐MIP POC sensor was further used in test of clinical plasma samples and compared with Simoa and ELISA. The GFAP concentrations range between 37 and 56 424 pg mL^−1^ (results tested with Simoa).^[^ ^58^ ^]^ In the ELISA tests, patient samples were diluted to 0.45 and 0.15 of the neat concentration, respectively. As for the detection with rGO/PDA‐MIP, all samples were diluted 1000 times to match the measuring range of the sensor. Considering the potential impact of fluid viscosity on measurement accuracy, we employed spike‐and‐recovery assays, as per the established norms in clinical diagnostic settings to ensure the reliability and reproducibility of the analytical results. The detailed test data from the three assays is shown in Tables S3 and S5 (Supporting Information). Figure 5C presents the comparison between the GFAP concentrations tested with Simoa, ELISA, and the developed rGO/PDA‐MIP sensor, where the Simoa test value was normalized representing 100% signal for each sample. The results obtained from our rGO/PDA‐MIP POC sensor displayed signal percentages ranging from 81.6% to 108.8%, with RSD value maintained below 7.1% for replicated samples. What is more, as shown in Figure 5D, the high consistency between the detection results from POC readout system and Simoa (k = 1.016) further proves the applicability of our sensor. (The detailed comparison between Simoa and ELISA test results was shown in Figure S5, Supporting Information) These findings underscored the sensor's capability for precise detection, even at trace levels of GFAP in bodily fluids. This capability is not only crucial for the early diagnosis of neurological disease but also plays a significant role in the ongoing management and treatment of the disease. The robustness and ease of operation position our rGO/PDA‐MIP POC sensor as a promising tool for integration into primary care and health screening programs.

## 3 Conclusion

This work reports the development of an innovative POC sensing system, tailored for the ultrasensitive quantification of GFAP in human plasma and CSF. The system utilizes a novel rGO/PDA‐MIP composite with abundance of imprinted cavities, demonstrating excellent performances including a broad linear response range, enhanced selectivity, consistent stability, and a remarkably low LoD. These characteristics are imperative for the clinical detection of GFAP, offering substantial improvements in diagnostic capabilities. The low‐cost and user‐friendly operation platform make it useful as a POC diagnostic tool for assessment of neurological diseases, especially suitable for implementation in primary healthcare environments, health screening initiatives, and regions with limited resources. It is hoped that the sensing platform could be further developed into miniaturized, wearable, wireless, and even self‐powered device in the future to monitor human health status with real‐time, wireless signal transmission, and convenient data visualization on mobile devices. Meanwhile, it is also expected to provide accurate and reliable information to build personalized health profiles and support remote clinical diagnosis. To realize the above objectives, some obstacles existed in current platform still need to be addressed, such as the suboptimal reusability, interference from the electrical and mechanical noise, and the limitation for wearable design due to the demand for test in solution containing redox labels. If with the above issues resolved, the device would realize great improvement and offer a more convenient, efficient, and accurate method for the detection and management of neurological diseases.

## 4 Experimental Section

### Reagents and Materials

Human GFAP (GFP‐H5143) was purchased from AcroBiosystems (USA). Vimentin (110‐10) was purchased from PeproTech (USA). Graphene oxide water dispersion (4 mg mL^−1^) was purchased from Graphenea (Spain). DA (CAS: 62‐31‐7) was purchased from Fisher Scientific (UK). D‐glucose (CAS: 50‐99‐7), uric acid (99%, CAS: 69‐93‐2), lactic acid (in H_2_O, ≥85%, CAS: 50‐21‐5), glycine (CAS: 56‐40‐6), sarcosine (≥98%, CAS: 107‐97‐1), cholesterol (CAS: 57‐88‐5), creatinine (CAS: 60‐27‐5), urea (CAS: 57‐13‐6), AP (≥98%, CAS: 7727‐54‐0), human serum albumin (≥95%, CAS: 70024‐90‐7), ascorbic acid (L‐ascorbic acid, 99%, CAS: 50‐81‐7), sodium dodecyl sulfate (SDS, ≥98.5%, CAS: 151‐21‐3), HCl (37 wt%, CAS: 7647‐01‐0), myoglobin (from human heart, ≥95%, CAS: 11080‐17‐4), phosphate buffer saline (PBS) tablets (1 tablet/200 mL, containing 0.01 m phosphate buffer, 0.0027 m potassium chloride and 0.137 m sodium chloride, pH 7.4), cytochrome c (from human heart, ≥95%, CAS: 9007‐43‐6), K_3_Fe(CN)_6_ (CAS: 13746‐66‐2), and K_4_Fe(CN)_6_·3H_2_O (CAS: 14459‐95‐1) were purchased from Sigma–Aldrich (UK). The mass fraction presented in percentage referred to the purity of the chemical. All chemicals were of analytical grade unless specified otherwise.

### Preparation of rGO/PDA‐MIP and NIP Composites

Initially, 1.5 µg of GFAP was introduced into 5 mL of PBS containing 2 mg of DA. The mixture was then stirred at room temperature for 1 h to facilitate the formation of monomer‐template complexes. Subsequently, 100 µL of GO water dispersion at a concentration of 4 mg mL^−1^ and 1.2 mg of the oxidant AP were added, continuously stirred for 24 h. This procedure simultaneously enabled the adhesion of DA‐template complex onto GO surface and the initiation of the DA monomer polymerization, resulting in the MIP composite with embedded templates. Then, the GFAP templates and any excess unreacted monomers were eluted by sequential washing through gentle agitation by hand with 5 mL elution solution (containing both 1 m HCl and 0.01 g mL^−1^ SDS) and deionized (DI) water, using a repeated cycle of centrifugation (5000 rpm, 20 min) and re‐suspension (washing product resuspended in DI water).

### Characterization of GO, rGO/PDA‐MIP and NIP Composites

Raman Spectroscopy analysis (Renishaw inVia, UK) was conducted using a 532 nm laser to examine molecular structural changes in the rGO/PDA‐MIP composite, using 10% laser power, 10 s exposure time, and three accumulations. XPS (Thermo Scientific K‐Alpha, USA) was employed for the surface chemical analysis with monochromatic Al Kα radiation at 150 W, referencing C1s and N1s lines at 284.2 and 399.7 eV, respectively. FTIR spectroscopy (PerkinElmer, UK) was used to compare the characteristic peaks of GO and rGO/PDA‐MIP composite, scanning in the 500–2000 cm^−1^ range. SEM (Hitachi 8230, UK) and TEM (FEI Talos F2000X) were utilized to investigate and compare the morphology characteristics of GO and rGO/PDA‐MIP composite under 20 and 200 kV accelerating voltages, respectively. TGA (Perkin‐Elmer 4000, UK) was executed to detect and compare weight loss during pyrolysis of GO and rGO/PDA‐MIP composite under nitrogen atmosphere from 20 to 800 °C at a heating rate of 20 °C min^−1^. BET analysis (Micromeritics Instrument Corporation adsorption analyzer, 3Flex) was conducted at 77 K to compare the pore size distribution of rGO/PDA‐MIP and NIP composites based on N_2_ adsorption and desorption isotherms.

### Electrochemical Measurements

Carbon‐based screen‐printed electrode (SPCE, DRP‐110, Metrohm) was served as the substrate electrode in this work. Ag electrode was integrated within the SPCE and used as reference for all electrochemical measurements. The modification of SPCE was achieved by drop‐casting 20 µL of rGO/PDA‐MIP composite dispersion (1 mg mL^−1^ in DI water) on working electrode, evaporated under 4 °C to mitigate the “coffee ring” effect. The modified electrodes were subsequently immersed in the electrolyte solution, including 0.01 m PBS at pH 7.4, 5 mm Fe(CN)_6_^3‐/4−^, and GFAP at varying concentrations. Electrochemical measurements were conducted following a 10‐min incubation with GFAP solutions.

The electrochemical behavior and performance of the rGO/PDA‐MIP‐modified electrodes were characterized using CV and DPV, respectively. CV measurements were performed with an electrochemical workstation (Autolab, PGSTAT204, Metrohm) at a scan rate of 50 mV s^−1^ across a potential range from −0.6 to +0.8 V, with step size at 2.44 mV. DPV measurements were conducted at a scan rate of 20 mV s^−1^ with a step potential of 10 mV, spanning a potential range from −0.5 to +0.8 V. The modulation amplitude, modulation time, and interval time were 0.025 V, 0.05 s, 0.5 s, respectively. DPV was repeated using both the commercial electrochemical workstation and a home‐designed readout system for control experiments.

### Human Samples

Clinical plasma samples were collected in the BIO‐AX‐TBI study from traumatic brain injury patients under a Health Research Authority approval (17/LO/2066). All participants gave informed consent per the Helsinki declaration. Venous bloods were sampled using ethylenediaminetetraacetic acid (EDTA)‐coated tubes. The samples were centrifuged at 2500 g at 4 °C, then transferred into 1.4 mL aliquots, and frozen at −80 °C for storage. The healthy control plasma for spiked test was sourced from Sigma‐Aldrich (UK), stored at 2–8 °C. The CSF sample (screened for GFAP) for spiked test was purchased from BIOIVT (UK), stored at −80 °C.

### Statistical Analysis

The GFAP concentrations in clinical plasma samples measured with Simoa were normalized to be 100% for each sample for the calculation of recovery rate. Error bars represent the standard deviation of the mean, with all data presented as mean ± SD. The sample size (n) for each analysis were contained in their figure legends. One‐way analysis of variance (ANOVA) was used to analyze the statistical differences. The *p*‐value <0.05 was considered to indicate statistically significant results, including *0.01 <*p* < 0.05, **0.001 <*p* < 0.01, ***0.0001 <*p* < 0.001, ****0.00001 <*p* < 0.0001, while ns (nonsignificant) was considered as no statistically significant difference. All data were analyzed with OriginPro 2023b software.

## Conflict of Interest

H.Z. has served at scientific advisory boards and/or as a consultant for Abbvie, Acumen, Alector, Alzinova, ALZPath, Amylyx, Annexon, Apellis, Artery Therapeutics, AZTherapies, Cognito Therapeutics, CogRx, Denali, Eisai, LabCorp, Merry Life, Nervgen, Novo Nordisk, Optoceutics, Passage Bio, Pinteon Therapeutics, Prothena, Red Abbey Labs, reMYND, Roche, Samumed, Siemens Healthineers, Triplet Therapeutics, and Wave, has given lectures in symposia sponsored by Alzecure, Biogen, Cellectricon, Fujirebio, Lilly, Novo Nordisk, and Roche, and is a co‐founder of Brain Biomarker Solutions in Gothenburg AB (BBS), which is a part of the GU Ventures Incubator Program (outside submitted work).

## Author Contributions

Y.L. performed conceptualization, visualization, investigation, formal analysis, methodology, data curation, validation, wrote the original draft and review, and performed editing. L.L. performed investigation, data curation, and visualization. L.S. performed investigation, validation, wrote the review, and performed editing. R.A., B.K., and K.X. acquired resources, wrote the review, and performed editing. E.T. acquired resources. L.X. and D.L. performed methodology, wrote the review, and performed editing. N.G.: Resources, writing‐review, and editing. A.H. performed investigation, data curation, formal analysis, and acquired resources. H.Z. and D.S. acquired funding, resources, and formal analysis, wrote the review, and performed editing. B.L. performed conceptualization, supervision, formal analysis, project administration, acquired resources, and funding, wrote the original draft, wrote the review and performed editing.

## Supporting information

Supporting Information

## Data Availability

The data that support the findings of this study are available from the corresponding author upon reasonable request.
