# Supplementary material for: An Ultrasensitive Molecularly Imprinted Point‐Of‐Care Electrochemical Sensor for Detection of Glial Fibrillary Acidic Protein
Source: Adv Healthc Mater. 2024 Sep 2;13(30):2401966. doi: 10.1002/adhm.202401966 (PMC11616259; doi:10.1002/adhm.202401966)
Supplement: Supplementary file 1 — Supporting Information [file ADHM-13-0-s001.docx]

**Supplementary Information**

Title: An Ultrasensitive Molecularly Imprinted Point-of-Care Electrochemical Sensor for Detection of Glial Fibrillary Acidic Protein

Yixuan Li^a^, Liuxiong Luo^b^, Lenart Senicar^a^, Rica Asrosa^a^, Burcu Kizilates^a^, Kaizhong Xing^c^, Elias Torres^d^, Lizhou Xu^e, f^, Danyang Li^g^, Neil Graham^h^, Amanda Heslegrave^i, j^, Henrik Zetterberg^i, j, k, l, m, n^, David J. Sharp^h, o^, Bing Li^a, h, o,^ *

^a^ Institute for Materials Discovery, Department of Chemistry, University College London, London, WC1E 7JE, UK

^b^ School of Materials Science and Engineering, Central South University, Changsha, 410083, PR China

^c^ Yusuf Hamied Department of Chemistry, University of Cambridge, Cambridge, CB2 1EW, UK

^d^ Graphenea Semiconductor, Paseo Mikeletegi 83, San Sebastián 20009, Spain

^e^ College of Biosystems Engineering and Food Science, Zhejiang University, Hangzhou, 310058, China

^f^ ZJU-Hangzhou Global Scientific and Technological Innovation Center, Zhejiang University, Hangzhou, 311215, China

^g^ Research Center, The Seventh Affiliated Hospital, Sun Yat-sen University, Shenzhen, 518107, China

^h^ Department of Brain Sciences, Imperial College London, London, W12 0BZ, UK

^i^ UK Dementia Research Institute at UCL, University College London, London WC1E 6BT, UK

^j^ Department of Neurodegenerative Disease, UCL Institute of Neurology, London WC1E 6BT, UK

^k^ Department of Psychiatry and Neurochemistry, Institute of Neuroscience and Physiology, the Sahlgrenska Academy at the University of Gothenburg, S-431 80 Mölndal, Sweden

^l^ Clinical Neurochemistry Laboratory, Sahlgrenska University Hospital S-431 80, Mölndal, Sweden

^m^ Hong Kong Centre for Neurodegenerative Diseases, Hong Kong 999077, China

^n^ Wisconsin Alzheimer’s Disease Research Center, University of Wisconsin School of Medicine and Public Health, University of Wisconsin-Madison, Madison, WI 53792, USA

^o^ Care Research & Technology Centre, UK Dementia Research Institute, London, W12 0BZ, UK

*Corresponding author.

1. mail: bing.li@ucl.ac.uk
2. **Design of miniaturized electrochemical readout system**

Fig. S1 (A) displays the POC electronic readout system for the detection of GFAP. A STM32F103C8T6 (STM32) microcontroller has been used to control the system. The entire system operates with a supply voltage at 5 V, with driving power and driving current of 30 mW and 30 mA, respectively. The amount of input power is relatively high to provide a maximum of 2 W, which is adequate when designing electrochemical measurement circuit, which at most has been shown to consume 1 W. This readout system can be powered by either standard 9 V batteries or via a micro-USB cable interfaced laptop (which is the case for this project), with the capability for measuring <0.1 μA current. The system offers easy operation with low dimensions (104 mm×83 mm), controlled by a key panel which includes functions of starting detection, setting start and stop potentials, setting step potential changes, displaying curves and peaks currents on screen, and uploading the digital results to laptop for further analysis. The system will be reset to the initial status once the test is completed.

The schematic in Fig. S1 (B) shows the workflow of the electrochemical detector. The STM32 can generate ramp voltage signals, where scan range and step size can be controlled by the key panel. The voltage is applied to the sensor, then the resulting current is converted back to voltage and processed into current. Fig. S1 (C) shows the main analog detection circuit, which includes four amplifiers (model OP07CP), labelled as U1A-U1D. These amplifiers act as the potentiostat, the current-to-voltage converter, the voltage inverter, and the filter and voltage shifter, respectively. The circuit also includes three ports R, W, and C, which would be connected to the screen-printed carbon electrode. The DAC receives signals from the STM32, which are stabilized by U1A and sent for the C port, generating DPV voltage signal. This signal will trigger the redox reactions on the surface of working electrode and the redox current would flow into the W port, which is then converted to a voltage signal by U1B, inverted by U1C, and then biased by U1D using a 0.5 V reference voltage. This signal is digitized by ADC of the STM32, completing the signal processing.

**Figure. S1.** Schematic of the miniaturized POC electrochemical readout system. (A) Appearance of the POC readout system; (B) The flow chart of the detection principle of the POC electrochemical readout system; (C) The main analog detection circuit in the POC electrochemical readout system.

1. **Optimization of rGO/PDA-MIP composite**

A systematic optimization has been carried out to identify the key factors that can affect the performance of rGO/PDA-MIP sensor, e.g., the mass ratio of templates to monomers and the incubation time of the modified electrodes in analyte solution. We have found that the mass ratio of templates to monomers plays a critical role in determining the number of imprinted cavities within the polymer matrix, which can significantly influence the Δ*I* during detections, and thereby affecting the sensitivity and the detection range [9]. The amount of DA monomers was fixed at 2 mg, with the addition of GFAP templates at 1.0, 1.5, 2.0, 2.5, and 3.0 μg, respectively. As illustrated in Fig. S2 (A), Δ*I*s were detected after the 10 mins incubation of fabricated MIPs modified electrodes in buffer containing 1 pg/mL GFAP target. It can be seen that the variations in the amount of GFAP template result in the notable changes in Δ*I*. With the initial rise in the amount of GFAP, an optimal Δ*I* peak appeared at 1.5 μg of template, indicating maximal recognition capability of the rGO/PDA-MIP composite towards the target molecules. This is attributed to the increased number of imprinting cavities, which owes to the increased template molecules added in fabrication process. However, with the continuous increase of template amount, the Δ*I* conversely decreased to even no distinguishable signal. This is due to that excessive templates would hamper monomer polymerization, in turn leading to the decreased number of binding cavities and even ineffective imprinting layer [10].

The impact of incubation time on the response of MIP sensor was also optimized. To avoid the cross-influence, the effect of different incubation time on MIPs fabricated with different amount of templates was also explored. Fig. S2 (B) investigated the change of Δ*I* versus the change of incubation period of MIPs modified electrode in 1 pg/mL GFAP solution. When the coated MIP was fabricated with addition of templates at 1.5 μg, the maximum increase in Δ*I* caused by target recognition has been observed, which saturated after the 10 min mark. This indicated that GFAP targets initially bind rapidly with the imprinting cavities, eventually at around 10 mins reaching a dynamic equilibrium between adsorption and desorption processes. When coated MIPs were fabricated with templates at 1.0 or 2.0 μg, Δ*I*s changed less with optimal incubation time slightly altered, but still showed similar increasing trend with increasing incubation time. Differently, as for the groups with MIPs prepared with templates at 2.5 and 3.0 μg, Δ*I*s kept low even if the incubation time increased, which demonstrated that MIPs fabricated under these conditions possessed no target recognition capability. This could be due to the excessive templates leading to the failure of monomers polymerization, which further led to the ineffective imprinting layer [11].


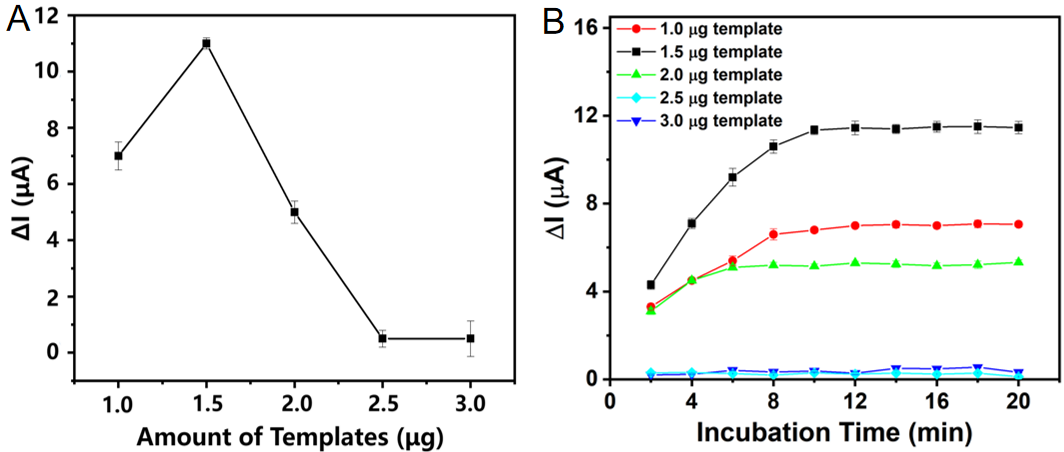


**Figure. S2.** Optimization of the rGO/PDA-MIP composite. (A) The value of ∆*I* versus the amount of template with fixed amount of monomer (2 mg); (B) The value of ∆*I* versus the incubation time of the electrode modified with MIPs prepared with different amounts of template (1.0, 1.5, 2.0, 2.5, 3.0 μg). Data are shown as the mean ± SD (n=3).

Except for the above, there are still other worth-exploring factors that might influence the sensing performance of fabricated MIP modified electrodes, such as polymerization time of template-monomer complex, the amount of oxidant, the amount of MIP modified on electrode surface, etc. These parameters deserve to be further explored in the future work with multivariate experimental design approach applied to avoid the cross-interference and get precise exploration results.

1. **Comparison of the proposed biosensor with other GFAP electrochemical sensors**

**Table S1.** Comparison of the proposed biosensor with other GFAP electrochemical sensors.

| **Sensing platform** | **Linear range** | **LoD** | **Ref** |
| --- | --- | --- | --- |
| Au@ZIF-8@rGO | 50.0–10000.0 fg/mL | 50.0 fg/mL | [1] |
| PEDOT:PSS/GiPEC | 1 pg/mL to 10 ng/mL | 281.7 fg/mL | [2] |
| PEDOT:PSS/PMMA | 10–1000 pg/mL | 3 pg/mL | [3] |
| PEI/Graphene | 1 pg/mL to 100 ng/mL | 1 pg/ml | [4] |
| HRP-Strep-btn-DAb-GFAP-CAb-MB-COOH-MBs | 0.24–31.6 ng/mL | 67 pg/mL | [5] |
| Anti-GFAP/L-cys/AuNPs/SPCE | 1.0–1000.0 pg/mL | 51.0 fg/mL | [6] |
| Polyamide/GFAP antibody | 7.81 to 2664 pg/mL | 14 pg/mL | [7] |
| PBASE/GFAP antibody/GFET | 2.3×10^2^ fg/mL to 2.3×10^2^ pg/mL | 20 fg/mL | [8] |
| GFAP antibody/Porous polyamide membrane | 7.81-2664 pg/mL | 14 pg/mL | [12] |
| rGO/PDA-MIP/SPCE | 1-10^6^ fg/mL | 483.6 ag/mL | This work |

Table S1 compares the performance of our proposed sensor with other existing GFAP electrochemical sensors. It could be seen that our sensor demonstrated a notably low LoD and a broad detection range.

1. **Effects of interferents on sensor response**

**Table S2.** Effects of interferents on sensor response (n=3).

|  | **Interferent** | **Matrix** | **Response (μA) (∆*I*)** | **Signal percentage (%)*** |
| --- | --- | --- | --- | --- |
| **Small molecule (MW＜1000 Da)** | **Urea** | Present in both CSF and plasma | 0.26 | 2.2 |
|  | **Creatinine** |  | 0.42 | 3.5 |
|  | **Glucose** |  | 0.39 | 3.3 |
|  | **Uric acid** |  | 0.30 | 2.5 |
|  | **Ascorbic acid** |  | 0.42 | 3.5 |
|  | **Lactic acid** |  | 0.45 | 3.8 |
|  | **Cholesterol** |  | 0.38 | 3.2 |
|  | **Glycine** |  | 0.35 | 2.9 |
|  | **Sarcosine** |  | 0.54 | 4.5 |
| **Macromolecule (MW＞5000 Da)** | **Vimentin** | Typically existed in plasma, but could be detected in CSF when central nervous system gets damaged | 0.24 | 2.0 |
|  | **Cytochrome C** | Typically existed in plasma, but could be detected in CSF under certain pathological situations involving cell death or apoptosis | 0.50 | 4.2 |
|  | **Myoglobin** | Typically existed in plasma, but could be detected in CSF when muscle is severely injured or damaged | 0.50 | 4.2 |
|  | **Albumin** | Present in both CSF and plasma | 0.41 | 3.4 |

^*^ The ratio of response from interferents to target analyte GFAP.

Table S2 shows the detailed response value from interferents, and also compares the signal strength detected from interferents and target analyte.

1. **Sensor performance test with commercial potentiostat**

To evaluate the precision of the POC readout system, the performance of the modified electrodes including sensitivity, selectivity, reproducibility, reusability, and stability were concurrently evaluated using the Autolab, as delineated in Fig. S3. As shown in Fig. S3 (A), the DPV peak currents of rGO/PDA-MIP-modified electrode similarly decreased with the increase of GFAP concentrations, spanning the detection range same at 10^3^-10^9^ ag/mL (1-10^6^ fg/mL) with a correlation coefficient of 0.996, whilst the rGO/PDA-NIP-modified electrode showed poor recognition capability (Fig. S3 (B)). The accuracy of this calibration plot had also been verified through triplicate measurements, resulting in RSD less than 5.7% for each concentration level.

What’s more, as shown in Fig. S3 (C), the ∆*I* measured by rGO/PDA-MIP sensor for GFAP detection was also significantly higher than that from other interferents under same concentration (1 pg/mL). The highest ∆*I* recorded from interferents, which was measured from vimentin, had been found only 5.2% of the signal for GFAP. ∆*I* assessed across five batches of the rGO/PDA-MIP-modified electrodes, as shown in Fig. S3 (D), presented excellent reproducibility with RSD less than 3.2%. As for the reusability depicted in Fig. S3 (E), the final obvious decrease in ∆*I* was observed to be the partial detachment or destruction of rGO/PDA-MIP composite coated on electrode surface during the repeated elution process. As shown in Fig. S3 (F), after three months of storage at room temperature, the initial response of the electrode modified with rGO/PDA-MIP remained 89.5%, indicating excellent long-term stability. The analogous detection results demonstrated that the home-designed electrochemical readout system had been endowed with comparable utility to commercial potentiostat.


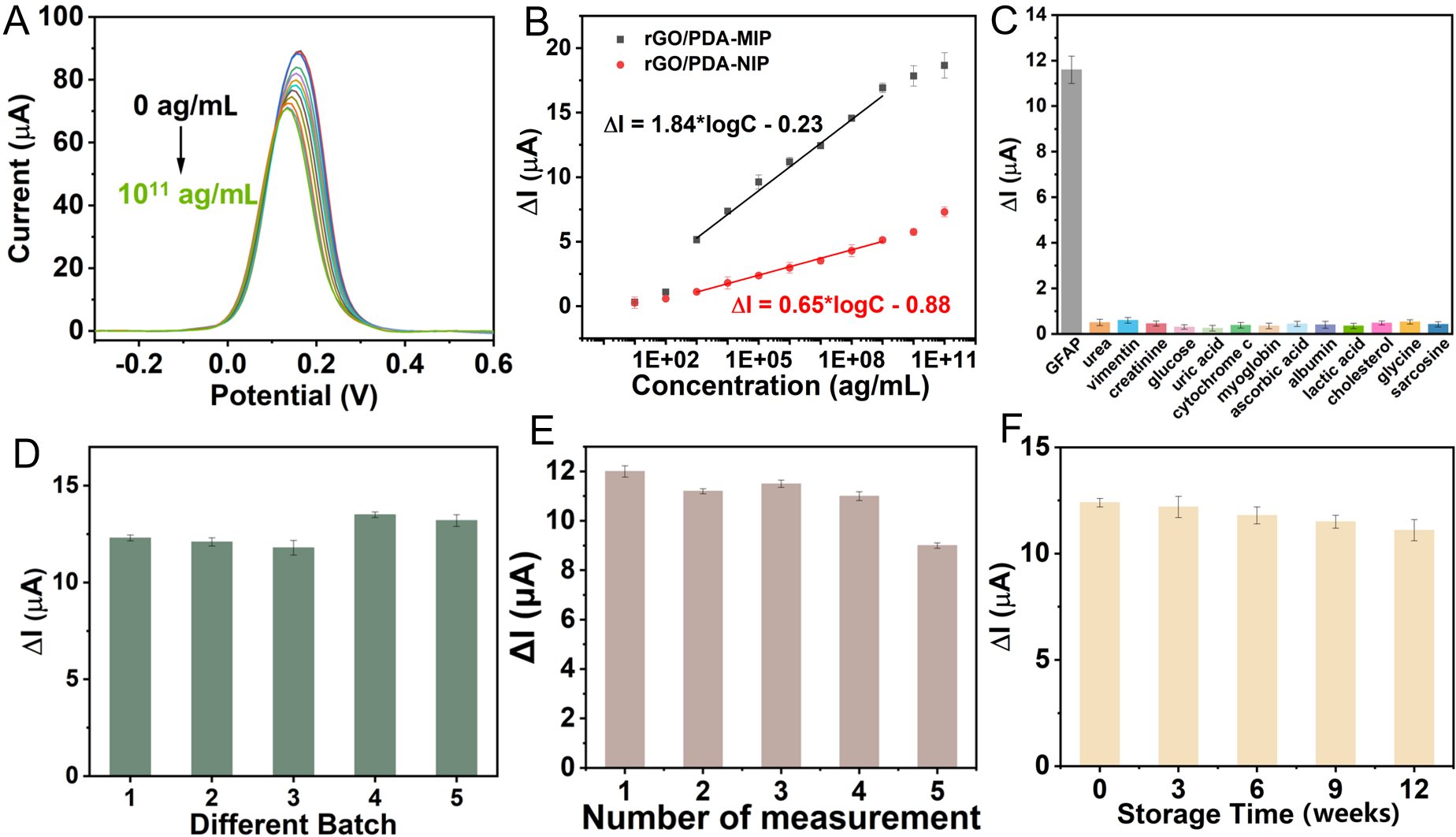


**Figure. S3.** Electrochemical performance of rGO/PDA-MIP-modified electrode for the detection of GFAP using commercial potentiostat. (A) The DPV responses of rGO/PDA-MIP-modified electrode to different concentrations of GFAP (0, 10^1^, 10^2^, 10^3^, 10^4^, 10^5^, 10^6^, 10^7^, 10^8^, 10^9^, 10^10^, and 10^11^ ag/mL) in the electrolyte; (B) Calibration plot of the ∆*I* values versus the log concentration of GFAP in the electrolyte tested with rGO/PDA-MIP and rGO/PDA-NIP-modified electrodes respectively; (C) The values of ∆*I* to the presence of GFAP at 1 pg/mL and other interferents in equivalent concentration; (D) The values of ∆*I* recorded with five batches of rGO/PDA-MIP-modified electrodes under same fabrication procedure; (E) The values of ∆*I* detected in five repeated measurements with rGO/PDA-MIP-modified electrode; (F) The values of ∆*I* tested in three months with rGO/PDA-MIP-modified electrode. Data are shown as the mean ± SD (n=3).

1. **Comparison of detection results from POC readout system and Autolab**

The comparison, illustrated in Fig. S4, shows an excellent consistency between the peak currents measured by the POC readout system and the Autolab, with a correlation coefficient of 0.982, proving that the POC readout system possesses reliability comparable to larger, more cumbersome commercial electrochemical workstation.

**
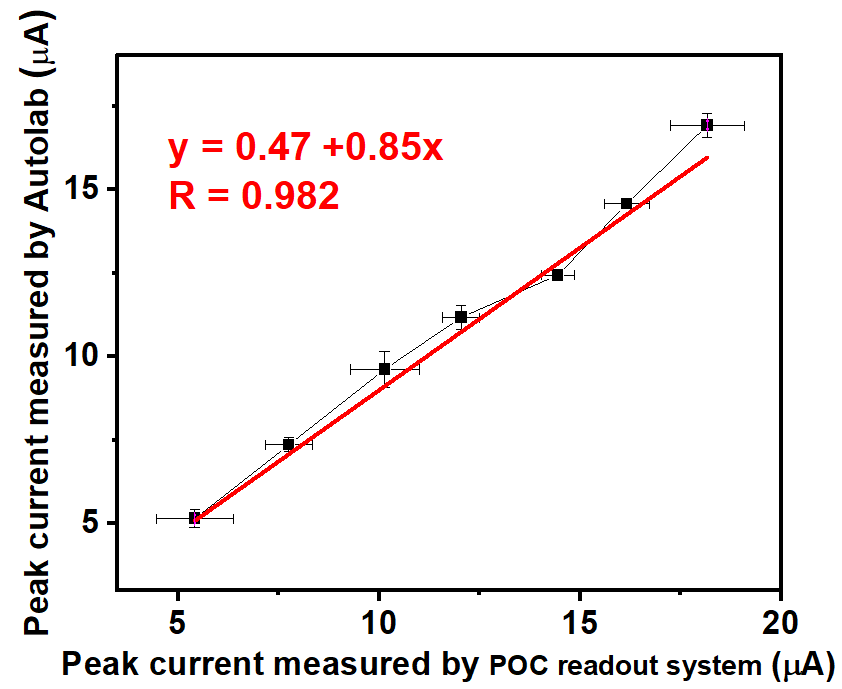
**

**Figure. S4.** The linear correlation of DPV peak currents measured by the POC readout system versus Autolab. The DPV peak currents refer to the values of ∆*I* of rGO/PDA-MIP-modified electrodes responded towards different concentrations of GFAP in the electrolyte. Data are shown as the mean ± SD (n=3).

1. **Determination of GFAP in clinical plasma samples with rGO/PDA-MIP**

**Table S3.** Determination of GFAP in clinical plasma samples with rGO/PDA-MIP (n=3).

|  | **Found^a^  (pg/mL)** | **Spiked^b^  (fg/mL)** | **Detected^c^  (fg/mL)** | **Recovery  (%)^d^** | **RSD (%)** |
| --- | --- | --- | --- | --- | --- |
| **Patient 1** | 56424 | 10000 | 62079.4 | 92.3 | 1.3 |
|  |  | 50000 | 109414.5 | 105.3 | 2.0 |
|  |  | 100000 | 155295.5 | 98.0 | 7.1 |
| **Patient 2** | 10108 | 5000 | 14643.0 | 95.4 | 4.8 |
|  |  | 10000 | 20997.5 | 108.8 | 2.8 |
|  |  | 20000 | 29632.9 | 95.3 | 3.6 |
| **Patient 3** | 36.7 | 10 | 47.4 | 101.8 | 2.9 |
|  |  | 30 | 62.4 | 88.3 | 5.5 |
|  |  | 50 | 79.9 | 81.6 | 4.7 |

^a^ The concentration of GFAP in clinical plasma samples tested by Simoa [8].

^b^ The concentration of formulated GFAP PBS solution.

^c^ The concentration of GFAP measured in the mixed solution with clinical sample diluted in the formulated GFAP PBS solution.

^d^ The ratio of (detected concentration-spiked concentration)/ (found concentration/dilution factor).

Table S3 shows the concentrations of GFAP in clinical plasma samples tested by this rGO/PDA-MIP sensor under spike-and-recovery assays, compared with the values tested through Simoa [8]. All samples were diluted 1000 times to match the measuring range of the sensor. The average recovery rate was 81.6-108.8% with the RSD value below 7.1% for replicate samples. These findings underscore the sensor's capability for precise detection, even at trace levels of GFAP in bodily fluid.

1. **Comparison of GFAP detection recovery rates in different matrices**

**Table S4.** Comparison of GFAP detection recovery rates in different matrices (n=3).

| **Matrix** | **Spiked concentration (pg/mL)** | **Detected concentration (pg/mL)** | **Recovery rate (%)** | **RSD (%)** |
| --- | --- | --- | --- | --- |
| **CSF from healthy control** | 0.08 | 0.074 | 92.5 | 9.5 |
|  | 0.4 | 0.416 | 104.0 | 2.3 |
|  | 2 | 2.036 | 101.8 | 0.3 |
|  | 10 | 9.952 | 99.5 | 1.1 |
|  | 50 | 54.811 | 109.6 | 5.4 |
| **Plasma from healthy control** | 0.08 | 0.084 | 105.0 | 9.6 |
|  | 0.4 | 0.389 | 97.3 | 1.7 |
|  | 2 | 1.854 | 92.7 | 8.7 |
|  | 10 | 9.233 | 92.3 | 3.0 |
|  | 50 | 48.010 | 96.0 | 1.5 |

Table S4 summarizes and compares the GFAP detection recovery rates in different matrices, including CSF and plasma from healthy controls.

1. **Determination of GFAP in clinical plasma samples with ELISA**

**Table S5.** Determination of GFAP in clinical plasma samples with ELISA (n=3).

|  | **ELISA#1  (pg/mL)** | **ELISA#2  (pg/mL)** | **Average (pg/mL)** |
| --- | --- | --- | --- |
| **Patient 1** | 19683 | 23516 | 21599.5 |
| **Patient 2** | 4831 | 4594 | 4712.5 |
| **Patient 3** | 467 | 283 (below LoD of the assay, discard) | 467.0 |

Table S5 shows the concentration of GFAP in clinical plasma samples tested by ELISA. Human GFAP ELISA Kit (ab223867) was used for GFAP detection in this assay. Both antibodies (the affinity tag labeled capture antibody and the reporter conjugated detector antibody) were diluted to working concentrations and mixed. Standards were reconstituted and a series of dilutions were prepared. The starting concentration was 80 ng/mL, further diluted 7 times with 1 in 3 steps. Patient samples were thawed and diluted to 0.45 and 0.15 of the neat concentration respectively. 50 μL of samples/standards were dispensed to wells, all conducted in triplicate. 50 μL of antibody mixture was added to all wells afterwards. The complex was sealed and incubated at room temperature for 1 h, then washed with 350 μL of the supplied wash buffer. Signal was generated after the addition and incubation with 100 μL of TMB development solution. The reaction stopped with addition of 100 μL of stop solution, with OD then read at 450 nm. The results were analyzed and graphed with GraphPad PRISM.

1. **Comparison of Simoa and ELISA test results**

Fig. S5 presented the detailed comparison of GFAP concentrations tested using Simoa and ELISA. The data of ELISA#2 from patient 3 has been discarded due to the lower detected value than assay LoD. Except for that, the concentrations tested by ELISA in both dilutions show comparable results with no significant difference, but do not match with the results measured by Simoa.

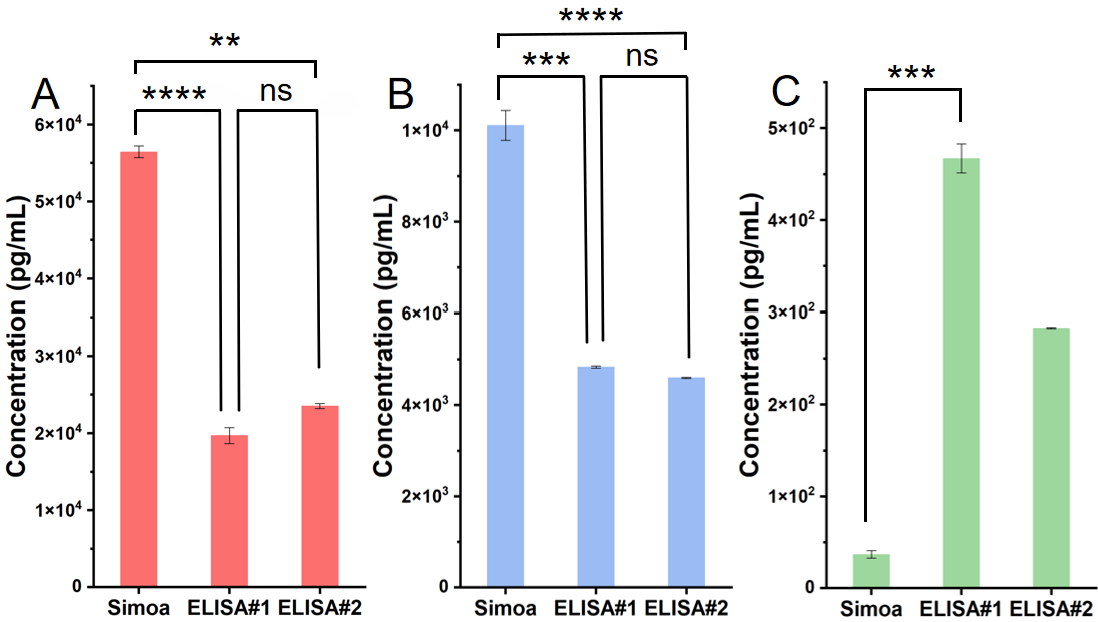


**Figure. S5.** Comparison of GFAP concentration in clinical plasma samples from (A) patient 1, (B) patient 2, and (C) patient 3 measured by Simoa and ELISA. Data are shown as the mean ± SD. *p < 0.05; **p < 0.01; ***p < 0.001; ****p < 0.0001; ns, not significant (n=3).

**References**

[1] M. Mehmandoust, E.E. Erk, M. Soylak, N. Erk, F. Karimi, *Ind. Eng. Chem. Res.* **2022**, *62* (11), 4532-4539.

[2] R. Salahandish, F. Haghayegh, S. Khetani, M. Hassani, A.S. Nezhad, *ACS Appl. Mater. Interfaces.* **2022**, *14* (25), 28651-28662.

[3] R. Salahandish, M. Hassani, A. Zare, F. Haghayegh, A. Sanati-Nezhad, *Lab on a Chip* **2022**, *22* (8), 1542-1555.

[4] S. Khetani, V. Ozhukil Kollath, V. Kundra, M.D. Nguyen, C. Debert, A. Sen, K. Karan, A. Sanati-Nezhad, *ACS Sens.* **2018**, *3* (4), 844-851.

[5] G. Ozcelikay, M. Gamella, M.A. Unal, K. Gucuyener, A. Montero-Calle, R. Barderas, J.M. Pingarrón, S. Campuzano, S.A. Ozkan, *Talanta* **2022**, *246*, 123526.

[6] G. Ozcelikay, F. Mollarasouli, M.A. Unal, K. Gucuyener, S.A. Ozkan, *Biosensors* **2022**, *12* (12), 1165.

[7] S. Shahub, K.C. Lin, S. Muthukumar, S. Prasad, *Biosensors* **2022**, *12* (12), 1095.

[8] L. Xu, S. Ramadan, O.E. Akingbade, Y. Zhang, S. Alodan, N. Graham, K.A. Zimmerman, E. Torres, A. Heslegrave, P.K. Petrov, *ACS Sens.* **2021**, *7* (1), 253-262.

[9] Z.M. Karazan, M. Roushani, *Talanta* **2022**, *246*, 123491.

[10] Y. Sun, H. Du, Y. Lan, W. Wang, Y. Liang, C. Feng, M. Yang, *Biosens. Bioelectron.* **2016**, *77*, 894-900.

[11] W. Liu, Y. Ma, G. Sun, S. Wang, J. Deng, H. Wei, *Biosens. Bioelectron.* **2017**, *92*, 305-312.

[12] S. Shahub, K.C. Lin, S. Muthukumar, S. Prasad, *Biosensors* **2022**, *12*(12), 1095.
